# Imaging and Microorganism Analyses of the Effects of Oral *Bifidobacterium breve* Intake on Facial Skin in Females: A Randomized, Double-Blind, Placebo-Controlled Study

**DOI:** 10.3390/nu17182976

**Published:** 2025-09-17

**Authors:** Yuriko Nishikawa, Chendong Xu, Shin Yoshimoto, Noriko Katsumata, Noriyuki Iwabuchi, Naotake Yanagisawa, Shigeo Koido, Miyuki Tanaka, Jin-Zhong Xiao, Daisuke Asaoka, Toshifumi Ohkusa, Nobuhiro Sato

**Affiliations:** 1 Department of Microbiota Research, Juntendo University Graduate School of Medicine, 3-3-1 Hongo, Ochanomizu KS Building 4F 405, Bunkyo, Tokyo 113-0033, Japanshigeo_koido@jikei.ac.jp (S.K.); j_xiao@morinagamilk.co.jp (J.-Z.X.);; 2 Innovative Research Institute, R&D Division, Morinaga Milk Industry Co., Ltd., Zama 252-8583, Japan; 3 Medical Technology Innovation Center, Juntendo University, Bunkyo 113-8421, Japan; 4 Department of Gastroenterology and Hepatology, The Jikei University Kashiwa Hospital, Kashiwa 277-8567, Japan; 5 Department of Gastroenterology, Juntendo Tokyo Koto Geriatric Medical Center, Koto 136-0075, Japan

**Keywords:** *Bifidobacterium breve* M-16V, gut microbiota, skin microbiota, probiotics, gut–skin axis

## Abstract

Background: Oral probiotic intake is suggested to have positive effects on skin. We aimed to elucidate the effects of oral *Bifidobacterium breve* M-16V intake on skin by analyzing facial images, the skin myco/microbiota, and the gut microbiota. Methods: We conducted a randomized, double-blind, placebo-controlled study in Japan. Healthy women aged over 30 years were randomly allocated to either the *B. breve* (1 × 1010 colony-forming units (CFU)/sachet, two sachets daily) or the placebo group and consumed the corresponding study food for 12 weeks. Facial images were taken at weeks 0, 4, 8, and 12 using VISIA evolution. Stool and skin samples were collected at weeks 0 and 12. The primary outcome was the change in the total VISIA score from baseline. Results: A total of 120 females aged 30–79 years were assigned to the *B. breve* (*n* = 59) or placebo (*n* = 61) group. The total VISIA score worsened in the placebo group at week 8 (*p* = 0.029) but not in the *B. breve* group. Compared with that of the placebo group, the VISIA brown spot score of the *B. breve* group improved at weeks 4 (*p* = 0.013) and 8 (*p* = 0.041). The VISIA pore score improved at weeks 4 (*p* = 0.013), 8 (*p* = 0.041), and 12 (*p* = 0.004) within the *B. breve* group. Genus-level analysis of the gut microbiota revealed a significant increase in *Blautia* abundance in the *B. breve* group. The frequency of adverse events was not different between the groups. Conclusions: Oral *B. breve* M-16V administration may suppress skin deterioration, including the appearance of brown spots, on the faces of adult females.

## 1. Introduction

The relationship between the gut microbiota and skin has attracted much attention. In general, the condition of the gut affects the skin, but its mechanism is still unclear. Recent studies have reported dysbiosis of the gut microbiota in individuals with inflammatory skin diseases, such as atopic dermatitis, acne vulgaris, and psoriasis vulgaris [1]. Intriguingly, the exacerbation of these diseases is also suggested to be associated with dysbiosis of the skin microbiota. The impact of the gut microbiota on skin homeostasis, as well as the skin microbiota, can be significant.

In recent years, many studies have examined the efficacy of oral probiotics in addition to topical agents in the pursuit of alleviating skin conditions. Studies investigating skin changes induced by the oral administration of probiotics have reported the amelioration of skin conditions, including the suppression of inflammation and reductions in photoaging effects. For example, oral intake of *Bifidobacterium breve* M-16V suppressed atopic dermatitis symptom scores in adult patients [2] and reduced skin scores among children with milk allergy [3]. Additionally, the oral administration of probiotics, such as *B. breve*, *Bifidobacterium longum*, *Lactobacillus plantarum*, and *Lactobacillus paracasei*, has been reported in several studies to have immunostimulatory effects on the skin, to inhibit the deterioration of skin barrier function, or to have antiphotoaging effects on the skin [4,5,6,7]. Furthermore, reports indicate that consuming probiotics orally can alter the skin microbiota. For example, in a trial involving children aged 3 to 36 months with atopic dermatitis, changes in skin microbiota were observed in the group that consumed *Lacticaseibacillus rhamnosus* for 12 weeks [8]. Higher levels of *Prevotella*, *Veillonella*, and *Ralstonia*, as well as lower levels of *Stenotrophomonas* and *Microbacterium*, were reported in this group. Another study of patients with acne vulgaris reported that 12 weeks of *L. plantarum* intake increased skin microbiota diversity and reduced the presence of *Propionibacterium* and *Corynebacterium* [9]. Among probiotic agents, *B. breve* M-16V has been suggested to have an anti-inflammatory effect on the skin and has been used in previous studies to treat both infants and adults with excellent safety. In this study, we aimed to investigate the changes in facial skin conditions, the skin and gut microbiota, and the skin mycobiota in the same subjects to elucidate the effects of oral intake of *B. breve* M-16V on skin conditions from both bacteriological and dermatological points of view. As estrogen levels are thought to be one of the factors affecting skin conditions [10], the study was conducted exclusively in female participants. In many previous trials related to skin health, subjective improvements in skin condition have been reported, even in the placebo group. Based on these results, this trial included subjective evaluations, dermatologist-based evaluations, and machine-based analytical evaluations.

## 2. Materials and Methods

### 2.1. Study Design

We conducted a randomized, double-blind, placebo-controlled study at a single center in Tokyo, Japan, from September 2021 to January 2022. The protocol was approved by the Japan Conference of Clinical Research on 15 July 2021. This study was registered with the University Hospital Medical Information Network (UMIN000045596). The participants were fully informed of the content of the research in advance, and all of them provided written informed consent prior to the study. All procedures were conducted in accordance with the Declaration of Helsinki.

### 2.2. Participants

The inclusion criterion was healthy women over 30 years old at enrolment. The exclusion criteria were as follows: (1) participants who regularly used cosmetic products or foods that affect skin conditions (excluding those who could discontinue the products throughout the study period), (2) participants who had skin diseases, had abnormal skin conditions requiring treatment, or suffered from diseases affecting the skin condition, (3) participants with severe allergic diseases or those who may develop such diseases during the study period, (4) participants with serious diseases or those with a history of serious diseases, (5) participants with a history of drug allergy or severe food allergy, (6) participants who regularly used foods, medicines, supplements, etc., that affect the intestinal microbiota (consumption for 4 or more days per week was considered regular use), (7) participants who were heavy drinkers, (8) participants who had a smoking habit of more than 20 cigarettes per day, and (9) participants who were pregnant, planned to become pregnant during the study period, or were breastfeeding.

### 2.3. Randomization and Masking

The participants were assigned to either the *B. breve* group or the placebo group according to the random assignment table that was generated by a person responsible for allocation after written informed consent was obtained. Computer-generated random number tables were used for randomization. Stratified randomization by mean age at menopause was performed for individuals under 50 years of age and over 50 years of age, which were considered allocation factors. This method was used because estrogen levels, which change significantly before and after menopause, are among the factors affecting the condition of the skin. We used a value of 50.5 years for the average age of menopause in the Japanese population, as reported by Tamada et al. [11], whose data were also used by the Ministry of Health. The table was stored in a sealed opaque envelope until all the data were collected. The test foods could not be distinguished by their packaging, taste, color, or odor. All the participants, the investigator, and the laboratory members who conducted the stool and skin sample analyses were blinded to the assigned groups.

### 2.4. Procedures

The participants were administered either probiotics or a placebo. The probiotics were administered as sticks containing 1 × 10^10^ CFUs of *B. breve* M-16V with maltodextrin as the excipient, and two of these sticks were consumed daily. The placebo powder contained maltodextrin only. The test foods were supplied by Morinaga Milk Industry Co., Ltd. (Tokyo, Japan). All participants were asked to continue using their skincare products and maintain their dietary habits, in addition to completing a diary to record the daily intake of the study food and changes in their skin and body conditions.

For the assessments of skin properties, participants underwent skin imaging, a skin examination, and questionnaires at baseline and at 4, 8, and 12 weeks. We evaluated the skin surface by using the VISIA Evolution system (Canfield Scientific, Fairfield, NJ, USA) for imaging analysis. The device was used to measure the number and extent of multiple features on and below the skin surface that are difficult to identify by the naked eye. The results of the imaging evaluation are displayed as scores (0–100; lower scores indicate a better skin condition) that reflect the number of instances of each feature and their severities. The participants were asked to wash their face and remain seated in an air-conditioned room for 20 min for acclimatization prior to the skin imaging and examination.

During the skin examination of the face, a physician evaluated the degree of symptoms for dryness and erythema on a 5-point scale, from 0 (no symptoms) to 4 (significant symptoms). The extent of wrinkles on the outer corner of the eyes was evaluated based on the guidelines of the Society of Cosmetic Chemists of Japan (8 grades from 0: no wrinkles to 7: distinct deep wrinkles).

In addition to the background data collected at baseline, the participants were asked to rate their skin condition at baseline and at 4, 8, and 12 weeks via a questionnaire. The condition of the skin was evaluated by the VAS method; specifically, skin glossiness, firmness, texture, dullness, spots, dark circles, redness, pores, dryness, wrinkles, tightness after washing, roughness, lip conditions, nail conditions, and hair conditions were quantified on a scale of 0–100.

For the skin and fecal microbiota/mycobiota analyses, skin samples and fecal samples were collected by the participants at home at baseline and at week 12. Fecal samples were collected using brush-type fecal collection kits with a preservative solution (Techno Suruga Laboratory Co., Ltd., Shizuoka, Japan). Skin samples for microbiota/mycobiota analyses were collected by using the MySkin tape striping kit (MySkin Co., Ltd., Tokyo, Japan) before the participants washed their face.

### 2.5. Outcomes

The primary outcome of this study was the change in the total VISIA score at weeks 4, 8, and 12 from baseline. The secondary outcomes were changes in VISIA subscores (reflecting brown spots, pores, porphyrins, red areas, spots, texture, UV spots, and wrinkles), the gut microbiota, the skin myco/microbiota, skin examination results (reflecting dryness, erythema, and wrinkles), and participants’ subjective evaluations. Adverse events were recorded in patient diaries or self-reported by participants.

Adverse events were graded by the study clinician according to severity based on NCI-CTCAE version 4.0 and relatedness to the study medication. All participants who received one dose of the study food were included in the safety analyses. In addition to having the participants record their intake of test foods in their diaries, we checked the amount of remaining test foods at the end of the trial to confirm the amount consumed.

### 2.6. Fecal DNA Preparation and Microbiota Analysis

Fecal DNA was prepared and the microbiota was analyzed as described previously [12]. Briefly, DNA was extracted from the fecal samples, and the purified DNA was suspended in 2000 µL of Tris–EDTA buffer (pH 8.0). PCR amplification and DNA sequencing of the V3–V4 region of the bacterial 16S rRNA gene were performed on an Illumina MiSeq instrument (Illumina, San Diego, CA, USA). After the removal of phiX reads from sequences aligned with Genome Reference Consortium human build 38 (GRCh38) and raw Illumina paired-end reads, the remaining sequences were analyzed using the QIIME2 software package version 2017.10 (https://qiime2.org/ (accessed on 10 December 2017)). DADA2 was used to remove potential chimeric sequences, followed by trimming of 30 and 90 bases from the 3′ region of the forwards and reverse reads, respectively. Taxonomic classification was performed using the naive Bayes classifier trained on Greengenes13.8 with a 99% threshold of operable classification units for full-length sequences. Alpha diversity was calculated using QIIME2 software, and R software (version 4.3.0) was used for the principal coordinate analysis (PCoA) based on the Jensen–Shannon distance (JSD).

### 2.7. Analysis of the Skin Microbiota and Mycobiota

DNA from the skin samples was extracted as described previously [13]. For the skin microbiota analysis, demultiplexed sequence data were analyzed using QIIME2 version 2022.2. All paired-end reads were trimmed to remove primer sequences with Cutadapt (via q2-cutadapt). Filtering of noisy sequences, error correction, removal of chimeric sequences/singletons, and the creation of amplicon sequence variants (ASVs) were performed by using DADA2 (via q2-dada2). Taxa were assigned to ASVs by using the q2-feature-classifier classify-sklearn naive Bayes taxonomy classifier trained on the SILVA database (release 138; 99% OTU reference sequences), with only the region of interest used for the analysis. The samples were rarefied to 24,000 sequences per sample (via the q2 feature table).

The skin mycobiota analysis was performed using the following methods. Each pair of reads was merged via USEARCH, which produced a single, fused read. The primer sequences were removed by Cutadapt. The reads where no primer region was detected were discarded. Low-quality reads were filtered with USEARCH (fastq_maxee parameter: 0.5). Reads less than 200 bp in length were also excluded. The resulting data were analyzed using QIIME1 version 1.9.0. Chimeric sequences were detected using USEARCH61 of the QIIME package and removed. OTU picking and taxonomic assignment were performed using QIIME. We performed open-reference OTU picking with an OTU threshold of 0.97 using the UNITE database (release s_28.06.2017) as the reference. The taxonomy of each OTU was assigned by UCLUST contained in the QIIME package. Based on the resulting OTU table with the assigned taxa, the abundance of fungal species in each sample was calculated.

### 2.8. Statistical Analysis

No previous studies have investigated the effects of oral probiotics on skin conditions using VISIA. In this study, we assumed an effect size of 0.5, a power of 0.80, and a significance level of 0.05, and the required sample size was 128. Considering discontinuation and dropout, we targeted 130 patients. Once data collection was complete, all the data were fixed before the groups were revealed. The primary endpoint was the difference in changes in VISIA scores from baseline between the 2 groups. Changes from baseline were compared between the probiotic and placebo groups by using the Wilcoxon rank-sum test. Moreover, intragroup changes in values between baseline and after the intervention were tested using the Wilcoxon signed-rank test. The primary analyses included the full analysis set, and those who received at least one dose of the study food were included in the safety analysis. Statistical analysis was performed by using the SAS software version 9.4 (SAS Institute, Cary, NC, USA), with the significance level set at *p* < 0.05. Intergroup differences in the fecal and skin microbiota and skin mycobiota at the phylum and genus levels were analyzed via the Wilcoxon rank-sum test. Alpha diversity indices were evaluated using the Wilcoxon rank-sum test. Differences in gut and skin microbiota and skin mycobiota profiles between the two groups were analyzed via principal coordinate analysis (PCoA). For multivariate analysis, permutational multivariate analysis of variance (PERMANOVA) was used to test the variation in microbiota and mycobiota composition explained by each factor. Correlations between the change in the VISIA score for brown spots and the fecal and skin microbiota changes and skin mycobiota changes were analyzed by calculating Spearman’s rank correlation coefficients. Statistical analyses were performed by using the R software (version 4.2.0) and IBM SPSS Statistics (version 28.0; Chicago, IL, USA), and a *p*-value of <0.05 was considered to indicate a significant difference.

### 2.9. Role of the Funding Source

This study was conducted at a joint research department between the Juntendo University Graduate School of Medicine and Morinaga Milk Industry Co., Ltd. Morinaga Milk Industry Co., Ltd., had no role in study design, data collection, data analysis, data interpretation, or writing of the report.

## 3. Results

**Figure 1:**
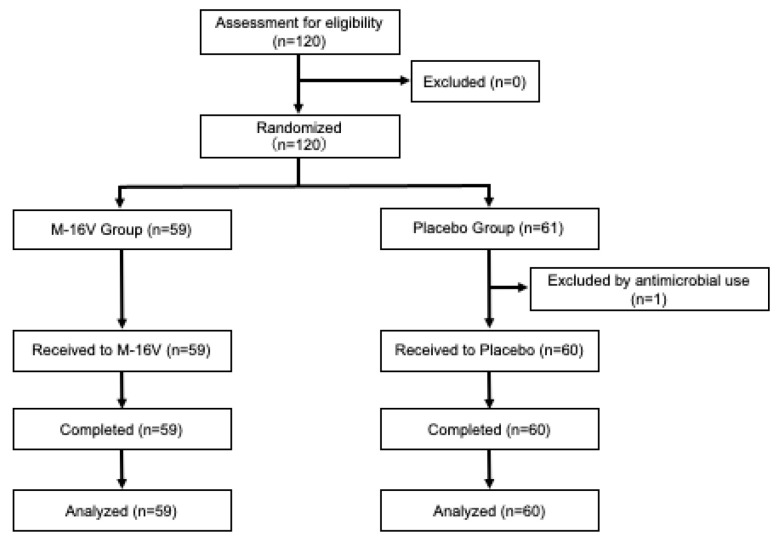
Flow diagram of participants was summarized according to the Consolidated Standards of Reporting Trials (CONSORT) and shows the numbers of participants who were randomized, lost to follow-up, and analyzed by the treatment group.

In this study, 120 women between the ages of 30 and 79 years were recruited from 1 September to 4 October 2021 and randomly assigned to two groups. As the skin condition is significantly affected by climate, we aimed to minimize the recruitment period to avoid uncertainty as to whether the skin changes were due to the study food or the climate. As a result, the recruitment of subjects was halted after the indicated period, at which point 120 participants had been enrolled, as the climate in Tokyo was predicted to change significantly from this point. Figure 1 shows the participant flow diagram. One participant in the placebo group was excluded because of the use of antibiotics prior to the consumption of the study food, and 119 participants completed the study (59 participants in the *B. breve* group and 60 participants in the placebo group). Details of the subjects’ background and characteristics are shown in Table 1. No significant differences in baseline background characteristics were observed between the two groups.

**Figure 2:**
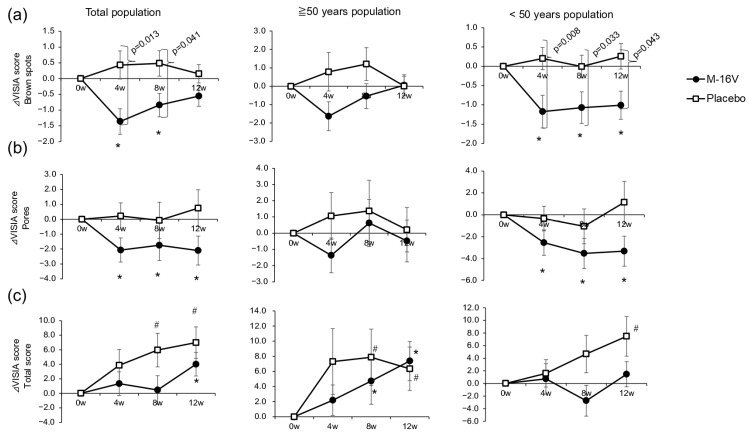
Changes in VISIA scores from week 0. (**a**) Brown spot scores, (**b**) pore scores, and (**c**) total scores. Comparisons between groups were made by using the Wilcoxon rank-sum test, and the Wilcoxon signed-rank test was used for each group for comparison with week 0 prior to ingestion. *p* < 0.05 was considered to indicate a statistically significant difference (* *p* < 0.05 vs. baseline for the M-16V group; ^#^ *p* < 0.05 vs. baseline for the placebo group).

Regarding the skin imaging results, the brown spot, pore, and total scores are shown in Figure 2. Since VISIA scores are higher when more of a particular feature is detected, a lower score indicates a better skin condition. Each VISIA value was compared between groups and within groups at each time point. The VISIA brown spot score significantly decreased from baseline in the *B. breve* group at 4 (median change: −1.00; IQRs −2.00 to 0.41; *p* = 0.001; r = −0.41) and 8 (median change: −0.50; IQRs −1.75 to 0.63; *p* = 0.034; r = −0.28) weeks. Significant differences were also observed between the two groups at week 4 (*B. breve*: median change: −1.00; IQRs −2.00 to 0.41; placebo: −0.05; IQRs −0.97 to 1.20; *p* = 0.013, r = −0.23) and week 8 (*B. breve*: median change: −0.50; IQRs −1.75 to 0.63; placebo: −0.06; IQRs −1.06 to 1.22; *p* = 0.041, r = −0.19), with the *B. breve* group having significantly lower scores. In particular, individuals under 50 years of age from the *B. breve* group presented significantly lower scores at week 4 (*B. breve*: median change: −1.07; IQRs −1.80 to −0.18; placebo: −0.03; IQRs −0.91 to 1.03; *p* = 0.008; r = −0.32), week 8 (*B. breve*: median change: −0.73; IQRs −1.74 to −0.08; placebo: −0.11; IQRs −1.04 to 1.11; *p* = 0.033; r = −0.26), and week 12 (*B. breve*: median change: −0.68; IQRs −1.95 to 0.60; placebo: 0.069; IQRs −0.90 to 1.12; *p* = 0.043; r = −0.24; Figure 2a).

Compared with those at baseline, the VISIA pore scores significantly decreased in the *B. breve* group during the study period (week 4: median change: −1.80; IQRs −4.88 to 0.95; *p* = 0.013; week 8: −1.63; IQRs −4.23 to 1.29; *p* = 0.041; week 12: −1.78; IQRs −4.32 to 1.03; *p* = 0.004), but no significant changes were observed in the placebo group. Similar results were observed in the population under 50 years of age (Figure 2b).

The total score increased significantly from the baseline value at weeks 8 (median change: 3.51; IQRs −3.58 to 13.7; *p* = 0.029) and 12 (median change: 6.96; IQRs −4.23 to 15.0; *p* = 0.002) in the placebo group, whereas the score in the *B. breve* group increased significantly from the baseline value only at week 12 (median change: 2.78; IQRs −3.43 to 10.8; *p* = 0.030). Individuals under 50 years of age in the placebo group presented a significant increase in the total score at week 12 compared with baseline, whereas no significant changes were observed in the *B. breve* group during the study period (Figure 2c). No marked change was observed in the other imaging scores (Supplementary Table S1).

The skin examination scores revealed no significant differences in dryness, erythema, or wrinkles at any follow-up time point between the *B. breve* and placebo groups (Supplementary Table S2).

According to self-evaluation, skin showed overall improvements in terms of skin glossiness, firmness, texture, dullness, spots, dark circles, redness, pores, dryness, wrinkles, tightness after washing, roughness, lip conditions, nail conditions, and hair conditions in both groups, with no differences between the groups (Supplementary Table S3).

**Figure 3:**
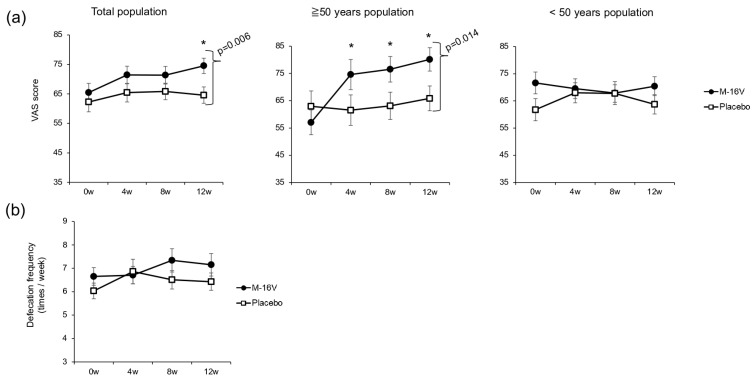
Bowel movement assessment. (**a**) VAS scores and (**b**) defecation frequency. Comparisons between groups were made by using the Wilcoxon rank-sum test, and the Wilcoxon signed-rank test was used for each group for comparison with week 0 prior to ingestion. A defecation VAS score of 0 represents the worst condition, while that of 100 represents the best condition. *p* < 0.05 was considered to indicate a statistically significant difference (* *p* < 0.05 vs. baseline for the *B. breve* group).

The results of the stool assessment are shown in Figure 3. The VAS score for defecation was significantly higher at week 12 in the *B. breve* group than in the placebo group. Among individuals over 50 years of age, the VAS scores for defecation were significantly higher in the *B. breve* group than the baseline score at all assessment points, and the score at week 12 was significantly higher than that in the placebo group (*p* = 0.014). Among individuals under 50 years of age, no significant intergroup or intragroup differences were observed (Figure 3a). However, no significant differences in defecation frequency were observed in either the total population or the among individuals under/over 50 years old (Figure 3b).

Regarding the fecal microbiota, no differences were observed in the alpha and beta diversities between the two groups at baseline and at week 12 (Supplementary Table S4 and Figure S1a), indicating that *B. breve* M-16V intake had no effect on the diversity of the gut microbiota. Similar results were obtained for the populations both under and over 50 years of age. The genus-level analysis revealed a significant increase in the *Blautia* abundance in the *B. breve* group (Supplementary Table S5). Among participants aged 50 years or older, significant increases in the abundances of Firmicutes at the phylum level and *Blautia* at the genus level were detected in the *B. breve* group.

Similar to the results for the fecal microbiota, no significant differences were observed in the alpha or beta diversity of the skin microbiota or skin mycobiota between the two groups in either the total population or when individuals were divided on the basis of age with a cutoff of 50 years (Supplementary Table S4 and Figure S1b,c). The analysis of the skin microbiota at the phylum and genus levels did not reveal significant differences between the two groups (Supplementary Table S6). In addition, no changes in the skin microbiota were detected in either the over-50 or under-50 age groups. No significant changes in the skin fungal composition at the phylum or genus level were observed between the two groups in the total population (Supplementary Table S6) or when individuals were divided on the basis of age with a cutoff of 50 years.

**Figure 4:**
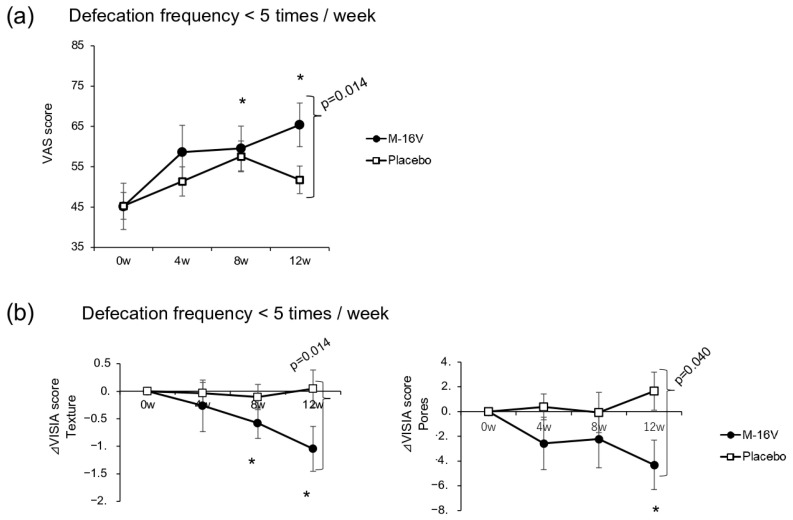
Bowel movement assessment and VISIA score change in a group with less defecation frequency. (**a**) Bowel movement assessment and (**b**) VISIA scores (*B. breve* group: *n* = 16; placebo group: *n* = 25). Comparisons between groups were made by using the Wilcoxon rank-sum test, and the Wilcoxon signed-rank test was used for each group for comparison with week 0 prior to ingestion. *p* < 0.05 was considered to indicate a statistically significant difference (* *p* < 0.05 vs. baseline for the *B. breve* group).

We examined both VAS scores for defecation and defecation frequency in those who had defecated less than five times per week at baseline. Although the defecation VAS scores were significantly improved in the *B. breve* group at weeks 8 and 12 compared with baseline, with a significant between-group difference at week 12 (*p* = 0.014), no significant difference in the defecation frequency was detected between the two groups (Figure 4a). Compared with that at baseline, the VISIA texture scores at weeks 8 and 12 were significantly lower in the *B. breve* group than in the placebo group (*p* = 0.014). A similar change was also observed for the VISIA pore score, with the *B. breve* group showing a significant reduction at week 12 compared with week 0 and a significant difference with the placebo group (*p* = 0.040; Figure 4b and Supplementary Table S7). Moreover, a significant increase in the abundance of *Faecalibacterium* and a significant decrease in the abundance of *Escherichia* in fecal microbiota were observed in the *B. breve* group compared with the placebo group at week 12 (Supplementary Table S8).

Adverse events occurred in 37.0% of the participants in total. The most common symptoms were dermatological problems, such as acne, flu symptoms, and gastrointestinal symptoms, including diarrhea. The occurrence of adverse events did not differ between the groups. All adverse events were mild and transient and were not related to the intake of the study foods.

## 4. Discussion

In this study, we evaluated the changes in the facial skin of adult women after 12 weeks of continuous intake of *B. breve* M-16V using imaging, skin examinations, an evaluation of subjective symptoms, and myco/microbiota analyses. Notably, the imaging evaluation revealed that the degree of brown spots and pores were decreased, and defecation-related symptoms improved during the study period according to subjective evaluation. A significant increase in the abundance of *Blautia* was identified in the fecal microbiota analysis, whereas no noteworthy changes were observed in the skin myco/microbiota analyses. Although this study was performed during the COVID-19 pandemic period, no participants were infected with the disease. Some of the study results have been presented at conferences, including the 18th Congress of the International Union of Microbiological Societies.

During September to January in Tokyo, skin conditions are generally expected to worsen due to dryness and cooler temperatures. In fact, symptoms and most VISIA scores, including the total VISIA score, in the placebo group tended to increase at each follow-up time point. On the other hand, in the *B. breve* group, the increase in VISIA scores, including the total score, was not observed. Notably, the VISIA brown spot score and pore score were significantly improved in the *B. breve* group. Many previous studies have suggested that probiotics, including *B. breve*, have anti-inflammatory effects, alleviating skin inflammation associated with diseases, such as atopic dermatitis and acne vulgaris. Interestingly, in this study, probiotic administration reduced the incidence of brown spots but not erythema. Because of infection control measures in Tokyo during the period of this study, the participants wore masks during environmental acclimatization. The possibility that the mask was an irritant to the skin and contributed to the redness of the cheeks should be considered. This point may be related to the findings that no significant differences were observed between groups in subjective evaluations using the VAS scale. While there are many reports of improvement in subjective evaluations in the placebo group in skin-related studies, it is also possible that the participants needed sufficient prior training in VAS.

The *B. breve* group showed a decrease in the VISIA brown spot score, and the change was particularly evident in the participants under 50 years of age. The VISIA brown spot score seemingly reflects several symptoms, such as solar lentigo, melasma, postinflammatory hyperpigmentation (PIH), and acquired dermal melanocytosis (ADM). Melasma is a brownish facial lesion worsened by the female hormone estrogen, in addition to friction and ultraviolet radiation [14]. It is particularly common in women in their 30s to 50s, who are prone to hormonal imbalances. PIH describes dark spots that can present in any age group and often disappear at 6–12 months [15]. In the case of solar lentigo, pigment fading may occur in the early stages [16]. Of the symptoms detected as brown spots, the fluctuating symptoms described above may be more readily alleviated by *B. breve* M-16V. Classifying brown spots in more detail is necessary in future studies.

Multiple processes are possible for oral probiotics to ameliorate brown spots. Hyperpigmentation is caused by increased melanogenesis or an abnormal distribution of the product melanin, resulting from the action of cytokines, inflammatory mediators, reactive oxygen species, keratinocytes, proteases, metalloproteinases, and the microphthalmia-associated transcription factor pathway [17]. Firstly, they may inhibit the accumulation of intestinal bacterial components or metabolites in the skin resulting from increased intestinal permeability and oxidative stress due to UV irradiation, both of which lead to skin barrier dysfunction and inflammation [18,19]. Oral probiotic intake, including *B. breve*, has previously been shown to suppress UV-induced barrier dysfunction and immunosuppression of the skin in animal models [20,21]. Additionally, the pathways related to female hormones should be considered. Liu et al. [22] reported a difference in the abundance of several bacteria in the gut between those with and without melasma. Considering that those bacteria are known to be involved in estrogen metabolism, one hypothesis that arises is that *B. breve* M-16V interacts with the intestinal environment of premenopausal women, potentially balancing female hormones. Normal levels of estrogen also stimulate proteins that maintain skin elasticity [23], which is one of the factors that make pores less visible. As described above, the oral intake of probiotics may contribute to the inhibition of melanin deposition in the skin via anti-inflammatory and hormone adjustment effects. Future studies are expected to elucidate the mechanism through the measurement of estrogen and hormone metabolism markers, such as β-glucuronidase in feces.

An improvement in the VISIA pore score was observed in the *B. breve* group in this study. Facial pores become recognizable mainly because of a reduction in skin elasticity, enlargement of hair follicles, or excessive sebum excretion [24]. The loss of skin elasticity is largely caused by UV radiation [25]. The oral administration of *B. breve* to chronically UV-irradiated mice countered these effects by suppressing changes in transepidermal water loss (TEWL), the skin water content, and epidermal thickening, suggesting its potential to improve skin elasticity [5]. How the gut microbiome is involved in sebum secretion remains unclear; however, a relationship between diet and sebum secretion has been suggested [26], and intestinal bacteria that affect digestion are potentially involved. The more obvious change in the VISIA pore scores observed in the younger group in this study may be because the age group with higher sebum secretion is 15–35 years [27], making such changes more detectable in individuals under 50. Little research has been conducted on the effects of probiotics on pores, but the results of this study indicate that future research, including studies of the relationships between the skin water content and barrier function and pores, is needed.

Women with abnormal bowel movements have many skin problems, including dry skin. This condition is thought to be due to the intestinal accumulation of substances with adverse effects on skin caused by constipation. In fact, the production of phenol, an intestinal metabolite, through the intake of symbiotics has been found to improve the skin condition [28]. The production of short-chain fatty acids (SCFAs) in the intestines promotes skin cell metabolism and improves skin barrier function [29]. In this study, no data were obtained to show that the improvement in defecation was directly related to an improvement in the skin in the overall population. However, in participants with infrequent defecation, the intake of *B. breve* M-16V led to an increase in the abundance of the genus *Faecalibacterium*, as well as improvements in the VISIA skin texture and the pore score. These findings suggest that *B. breve* M-16V may improve the intestinal environment through the increased production of butyric acid due to an increased abundance of species of the genus *Faecalibacterium*, which suppresses the production of harmful metabolites and increases the water content in the skin. Changes in fecal and serum metabolite levels including SCFA should be assessed in future studies.

Symptoms of defecation were alleviated according to subjective evaluation, especially among those aged over 50 years, but no significant change was observed in the frequency of bowel movements. The results of this study suggest that participants in this age group may have been aware of improvements in aspects other than the frequency of defecation. In addition, in this study, the number of defecations was calculated based on participants’ memory of the past month, which may not have been sufficient to accurately determine the frequency of defecation. Many subjects with no abnormal bowel movements may have been included in the study, which focused primarily on investigating the effects on the skin. Surveys on bowel movements are affected by memory bias, and future studies will require surveys based on constipation scores and accurate records.

This study performed a detailed analysis of the effects of the oral administration of probiotics on the gut microbiota, skin microbiota, and skin mycobiota of healthy adult women using samples from the same subjects. In this study, *B. breve* M-16V intake significantly increased the relative abundance of *Blautia* spp. in the gut microbiota of individuals aged over 50 years. *Blautia* spp. are some of the major bacteria in the human gut microbiota and mainly produce organic acids, such as acetic acid, lactic acid, and succinic acid. Although the efficiency of dietary fiber utilization varies, generally *Blautia* and *Bifidobacterium*, whose abundance decrease with age, are well known to use dietary fiber to proliferate [30]. Interestingly, a study of Japanese subjects suggested that *Bifidobacterium* and *Blautia* have a cross-feeding relationship [31]. *B. breve* also indirectly increases the abundance of *Blautia wexlerae* in animal models [32]. The increase in *Blautia* in this study with the oral intake of *B. breve* M-16V also suggests an interaction between these bacteria.

Regarding skin microorganisms, the skin microbiome analysis revealed no significant differences in alpha or beta diversity in this study. Previous studies on areas with skin lesions have reported inconsistent findings regarding the diversity of the skin microbiota [9,33,34]. In this study, the investigation was conducted on healthy skin, so changes in the skin microbiota were minimal, and it is possible that differences in diversity were not clearly apparent. Similar to the findings of Byrd et al. [35], *Cutibacterium* (*Propionibacterium*), *Corynebacterium*, and *Staphylococcus* were detected as major bacteria in the facial skin of healthy adults, and *Malassezia* was found to be the predominant skin fungus. Moreover, 12 weeks of *B. breve* M-16V administration did not significantly affect the skin microorganisms in healthy individuals. To our knowledge, this study is the first to simultaneously analyze changes in the gut microbiota, skin microbiota, and skin mycobiota of healthy adult subjects in response to intervention with oral probiotics. On the other hand, Yu et al. published a study in which probiotics were administered after doxycycline treatment to patients with rosacea. The combination of antibiotics and probiotics improved the facial skin condition, alleviated inflammation, reduced the skin microbiota diversity, and increased the gut microbiota heterogeneity. Additionally, a decrease in the expression of antibiotic resistance genes was also observed [33]. Clear investigations of the interaction between the gut environment and skin microorganisms will be necessary to determine the usefulness of oral probiotics for improving skin conditions.

One limitation of this study is that the VISIA algorithm is proprietary, and its results are not currently considered validated endpoints. Thus, its correlation with physiological measurements limits clinical interpretation. Nonetheless, the device has been used in clinical trials, and we believe that it can provide objective measurements of facial areas and visual indicators that are difficult to observe with the human eye. In future trials, it would be useful to measure physiological skin parameters, such as TEWL, skin elasticity, and epidermal thickness, alongside VISIA, and to perform more detailed analyses of blood hormone levels and gut metabolites, including hormone metabolism markers and SCFAs, to better understand the gut–skin axis. Overall, the advantages of this strain are not universal in this study, and the results of this study suggest that the benefits of probiotic intake may depend on factors such as age, lifestyle, medical history, and particularly, intestinal environment.

## 5. Conclusions

Twelve weeks of *B. breve* M-16V intake may improve brown spots and pores on the facial skin of women aged between 30 and 50 years.

## Figures and Tables

**Table 1:** Baseline characteristics of subjects in each group.

	M-16V *n* = 59	Placebo *n* = 60	*p*-Value ^a^
Age (years)	46.6 ± 12.3	47.2 ± 10.9	0.73
Height (cm)	158.7 ± 5.50	160.1 ± 4.90	0.19
Body weight (kg)	53.02 ± 6.79	53.18 ± 6.53	0.82
BMI (kg/cm^2^)	21.08 ± 2.72	20.75 ± 2.47	0.52
Allergy, n (%)	30 (51.7)	25 (41.7)	0.36
Birth history, n (%)	38 (65.5)	42 (70.0)	0.69
Menstruation, n (%)	31 (53.5)	33 (55.0)	0.87
Lotion use, n (%)	53 (89.8)	56 (93.3)	0.53
Beauty serum use, n (%)	32 (54.2)	40 (66.7)	0.19
Emulsion use, n (%)	35 (59.3)	37 (61.7)	0.85
Cream use, n (%)	31 (52.5)	28 (46.7)	0.58
Sunblock cream use, n (%)	47 (81.0)	49 (83.1)	0.81
Sunblock supplement use, n (%)	1 (1.8)	3 (5.6)	0.36
Yogurt intake habits, n (%) ^b^	24 (40.7)	21 (35.0)	0.57
Supplement intake habits, n (%) ^b^	17 (28.8)	20 (33.3)	0.69

Abbreviations: BMI: body mass index. The values are presented as the mean ± SD. ^a^ Continuous variables were statistically analyzed by *t*-test, ordinal variables by Wilcoxon rank-sum test, and categorical variables by Fischer’s exact test. ^b^ Number of people who are in the habit of taking it at least three days a week.

## Data Availability

The original contributions presented in this study are included in the article/Supplementary Material. Further inquiries can be directed to the corresponding authors.

## Supplementary Materials

The following supporting information can be downloaded at: https://www.mdpi.com/article/10.3390/nu17182976/s1. Table S1: VISIA score for each skin indicator; Table S2: Skin findings related to dryness, erythema, and wrinkles; Table S3: VAS score for subjective skin symptoms; Table S4: Alpha diversity of fecal and skin microbiota and skin mycobiota in M-16V and placebo groups; Table S5: Composition of the fecal microbiota in M-16V and placebo groups; Table S6: Composition of the skin microbiota and mycobiota in M-16V and placebo groups; Table S7: VISIA score for each skin indicator in a group with low defecation frequency; Table S8: Composition of the fecal microbiota with low defecation frequency in M-16V and placebo groups; Figure S1: Principal coordinate analysis (PCoA) plots of bacterial and fungal beta diversity based on the Bray–Curtis dissimilarity and Jaccard, unweighted UniFrac, and weighted UniFrac distances analysed by time points.
