# Supplementary material for: Imaging and Microorganism Analyses of the Effects of Oral Bifidobacterium breve Intake on Facial Skin in Females: A Randomized, Double-Blind, Placebo-Controlled Study
Source: Nutrients. 2025 Sep 17;17(18):2976. doi: 10.3390/nu17182976 (PMC12472256; doi:10.3390/nu17182976)
Supplement: Supplementary file 1 [file nutrients-17-02976-s001.zip › Supfiles/Nutrients_Supfiles.docx]

| Table S1.VISIA score for each skin indicator. | | |  |  |  |  |  |  |  |
| --- | --- | --- | --- | --- | --- | --- | --- | --- | --- |
|  |  | M-16V | Placebo | Between-groups | Within-group  *P*-value | | Difference between changes from baseline | | |
|  |  |  |  | *P*-value | M-16V | Placebo | M-16V | Placebo | *P*-value |
| Brown spots | Baseline | 21.6 (16.1-25.8) | 17.8 (14.3-22.1) | 0.009* | - | - | - | - | - |
|  | Week 4 | 20.0 (14.9-26.2) | 17.9 (14.8-23.9) | 0.365 | 0.001* | 0.905 | -1.00 (-2.00-0.41) | -0.05 (-0.97-1.20) | 0.013* |
|  | Week 8 | 20.8 (15.3-25.4) | 17.8 (14.6-22.2) | 0.122 | 0.034* | 0.603 | -0.50 (-1.75-0.63) | -0.06 (-1.06-1.22) | 0.041* |
|  | Week 12 | 20.7 (15.4-26.3) | 18.5 (15.4-22.2) | 0.080 | 0.095 | 0.483 | -0.44 (-1.81-0.99) | 0.10 (-0.90-1.40) | 0.090 |
| Pores | Baseline | 20.5 (14.9-30.2) | 17.7 (9.95-26.4) | 0.059 | - | - | - | - | - |
|  | Week 4 | 19.3 (12.3-29.7) | 17.9 (10.3-28.5) | 0.415 | 0.013* | 0.835 | -1.80 (-4.88-0.95) | 0.41 (-3.67-2.24) | 0.066 |
|  | Week 8 | 21.6 (12.3-29.2) | 17.1 (11.1-25.6) | 0.337 | 0.041* | 0.533 | -1.63 (-4.23-1.29) | -0.27 (-3.43-2.20) | 0.305 |
|  | Week 12 | 19.5 (12.6-29.3) | 17.3 (9.37-26.2) | 0.273 | 0.004* | 0.896 | -1.78 (-4.32-1.03) | -0.13 (-3.11-3.11) | 0.068 |
| Porphyrins | Baseline | 4.23 (2.71-9.61) | 4.34 (2.72-7.42) | 0.846 | - | - | - | - | - |
|  | Week 4 | 4.11 (2.67-8.30) | 4.61 (3.01-7.60) | 0.901 | 0.602 | 0.828 | -0.06 (-0.81-0.96) | -0.13 (-0.74-1.26) | 0.643 |
|  | Week 8 | 4.30 (2.59-8.11) | 3.84 (2.50-8.00) | 0.847 | 0.229 | 0.217 | -0.15 (-1.11-0.50) | -0.40 (-1.09-0.67) | 0.865 |
|  | Week 12 | 4.53 (2.99-9.07) | 4.49 (2.47-6.63) | 0.446 | 0.280 | 0.069 | -0.23 (-1.13-0.52) | -0.37 (-1.35-0.57) | 0.473 |
| Red areas | Baseline | 9.01 (8.34-10.6) | 9.30 (7.81-10.6) | 0.848 | - | - | - | - | - |
|  | Week 4 | 9.23 (8.34-10.3) | 9.19 (7.82-10.1) | 0.346 | 0.919 | 0.636 | -0.03 (-0.63-0.60) | -0.04 (-1.26-0.95) | 0.665 |
|  | Week 8 | 9.14 (8.20-10.6) | 9.25 (8.11-10.4) | 0.862 | 0.665 | 0.212 | 0.18 (-1.01-0.93) | 0.15 (-0.67-1.43) | 0.553 |
|  | Week 12 | 9.39 (8.07-11.2) | 9.64 (7.77-11.2) | 0.971 | 0.261 | 0.044* | 0.26 (-0.83-1.12) | 0.28 (-0.30-1.43) | 0.794 |
| Spots | Baseline | 26.5 (21.4-32.7) | 24.5 (20.6-31.0) | 0.199 | - | - | - | - | - |
|  | Week 4 | 27.6 (20.5-32.6) | 25.2 (18.7-33.0) | 0.316 | 0.922 | 0.849 | 0.10 (-2.37-2.08) | 0.13 (-2.48-2.74) | 0.811 |
|  | Week 8 | 26.7 (22.6-34.2) | 25.1 (20.8-28.8) | 0.217 | 0.966 | 0.241 | -0.16 (-2.03-1.68) | 1.04 (-1.47-2.69) | 0.355 |
|  | Week 12 | 27.3 (22.8-34.4) | 23.6 (20.2-30.8) | 0.132 | 0.165 | 0.237 | 0.42 (-1.97-3.00) | 0.55 (-1.06-2.11) | 0.652 |
| Texture | Baseline | 5.25 (3.65-8.19) | 5.86 (2.79-8.33) | 0.968 | - | - | - | - | - |
|  | Week 4 | 5.80 (3.43-8.93) | 6.13 (2.76-8.92) | 0.959 | 0.424 | 0.444 | -0.22 (-0.63-0.65) | 0.16 (-0.46-0.78) | 0.246 |
|  | Week 8 | 4.82 (3.56-8.55) | 5.98 (2.99-8.25) | 0.940 | 0.503 | 0.921 | -0.05 (-0.94-0.66) | 0.00 (-0.66-0.83) | 0.665 |
|  | Week 12 | 5.37 (3.47-7.90) | 5.79 (3.04-8.09) | 0.885 | 0.092 | 0.666 | -0.25 (-1.30-0.36) | 0.15 (-1.17-0.71 | 0.410 |
| UV spots | Baseline | 22.5 (16.6-29.2) | 20.2 (15.6-24.4) | 0.031* | - | - | - | - | - |
|  | Week 4 | 23.1 (17.3-28.5) | 23.3 (15.5-26.1) | 0.343 | 0.224 | 0.189 | -0.52 (-1.66-1.05) | 0.60 (-1.28-2.18) | 0.069 |
|  | Week 8 | 24.2 (17.2-28.4) | 22.1 (16.3-26.1) | 0.115 | 0.684 | 0.093 | 0.17 (-0.98-1.18) | -0.09 (-0.98-3.00) | 0.335 |
|  | Week 12 | 23.4 (19.1-29.6) | 20.9 (16.5-25.8) | 0.050 | 0.071 | 0.025* | 0.29 (-0.68-1.75) | 0.60 (-0.81-3.26) | 0.592 |
| Wrinkles | Baseline | 28.4 (19.7-38.6) | 33.5 (21.9-43.0) | 0.332 | - | - | - | - | - |
|  | Week 4 | 37.1 (28.0-43.5) | 35.0 (24.6-41.8) | 0.398 | <0.001* | 0.003* | 4.69 (0.15-9.34) | 2.78 (-0.48-5.86) | 0.099 |
|  | Week 8 | 32.4 (22.2-43.0) | 35.2 (24.6-42.0) | 0.490 | 0.002* | <0.001* | 2.52 (-1.58-6.03) | 3.63 (-0.35-9.37) | 0.375 |
|  | Week 12 | 34.7 (25.3-44.9) | 38.8 (28.6-46.7) | 0.351 | <0.001* | <0.001* | 4.92 (-0.29-9.38) | 5.27 (-0.96-12.9) | 0.779 |
| Total score | Baseline | 143.5 (130.0-167.0) | 136.7 (113.7-159.7) | 0.089 | - | - | - | - | - |
|  | Week 4 | 150.1 (121.5-173.0) | 142.6 (119.5-167.2) | 0.290 | 0.552 | 0.106 | 0.72 (-5.57-8.51) | 5.86 (-6.42-11.1) | 0.316 |
|  | Week 8 | 145.5 (124.9-166.8) | 147.2 (115.4-165.5) | 0.394 | 0.742 | 0.029* | 1.25 (-9.10-8.98) | 3.51 (-3.58-13.7) | 0.163 |
|  | Week 12 | 150.3 (129.5-175.5) | 147.6 (119.4-170.4) | 0.228 | 0.030* | 0.002* | 2.78 (-3.43-10.8) | 6.96 (-4.23-15.0) | 0.233 |
| Data are presented as the medians (interquartile ranges). A higher value for the VISIA score indicates a worse condition, while a lower value indicates a better condition. | | | | | | | | | |
| The VISIA total score was calculated by adding all eight items from brown spots to wrinkles for each subject. | | | | | | |  |  |  |
| The Wilcoxon rank sum test was used to analyze differences between groups. | | | | |  |  |  |  |  |
| Within-group p-value for each grpup was calculated by comparing against baseline using the Wilcoxon signed rank test. | | | | | | | |  |  |

| Table S2. Skin findings related to dryness, erythema, and wrinkles | | | | | | |  |  |  |  |  |  |  |  |  |  |  |
| --- | --- | --- | --- | --- | --- | --- | --- | --- | --- | --- | --- | --- | --- | --- | --- | --- | --- |
|  |  | week 0 | |  |  | week 4 | |  |  | week 8 | |  |  | week 12 | |  |  |
|  |  | M-16V, n (%) n=59 | Placebo, n (%) n=60 | *P*-value |  | M-16V, n (%) n=55 | Placebo, n (%) n=52 | *P*-value |  | M-16V, n (%) n=56 | Placebo, n (%) n=55 | *P*-value |  | M-16V, n (%) n=55 | Placebo, n (%) n=57 | *P*-value |  |
| Dryness | |  |  |  |  |  |  |  |  |  |  |  |  |  |  |  |  |
|  | 0 | 0 (0.0) | 0 (0.0) | 0.11 |  | 0 (0.0) | 0 (0.0) | 0.66 |  | 0 (0.0) | 0 (0.0) | 0.42 |  | 0 (0.0) | 1 (1.8) | 0.26 |  |
|  | 1 | 2 (3.4) | 4 (6.7) |  |  | 8 (14.6) | 6 (11.5) |  |  | 8 (14.3) | 2 (3.6) |  |  | 11 (20.0) | 6 (10.5) |  |  |
|  | 2 | 20 (33.9) | 27 (45.0) |  |  | 22 (40.0) | 20 (38.5) |  |  | 23 (41.1) | 27 (49.1) |  |  | 27 (49.1) | 29 (50.9) |  |  |
|  | 3 | 37 (62.7) | 29 (48.3) |  |  | 24 (43.6) | 26 (50.0) |  |  | 24 (42.9) | 25 (45.5) |  |  | 17 (30.9) | 18 (31.6) |  |  |
|  | 4 | 0 (0.0) | 0 (0.0) |  |  | 1 (1.8) | 0 (0.0) |  |  | 1 (1.8) | 1 (1.8) |  |  | 0 (0.0) | 3 (5.3) |  |  |
| Erythema | |  |  |  |  |  |  |  |  |  |  |  |  |  |  |  |  |
|  | 0 | 0 (0.0) | 0 (0.0) | 0.69 |  | 0 (0.0) | 0 (0.0) | 0.20 |  | 0 (0.0) | 1 (1.8) | 0.10 |  | 0 (0.0) | 0 (0.0) | 0.52 |  |
|  | 1 | 7 (11.9) | 2 (3.3) |  |  | 12 (21.8) | 9 (17.3) |  |  | 15 (26.8) | 8 (14.6) |  |  | 25 (45.5) | 24 (42.1) |  |  |
|  | 2 | 28 (47.5) | 34 (56.7) |  |  | 24 (43.6) | 19 (36.5) |  |  | 19 (33.9) | 16 (29.1) |  |  | 22 (40.0) | 20 (35.1) |  |  |
|  | 3 | 23 (39.0) | 24 (40.0) |  |  | 19 (34.6) | 22 (42.3) |  |  | 2 (3.6) | 3 (5.5) |  |  | 5 (9.1) | 11 (19.3) |  |  |
|  | 4 | 1 (1.7) | 0 (0.0) |  |  | 0 (0.0) | 2 (3.9) |  |  |  |  |  |  | 3 (5.5) | 2 (3.5) |  |  |
| Wrinkles | |  |  |  |  |  |  |  |  |  |  |  |  |  |  |  |  |
|  | 0 | 6 (10.2) | 5 (8.3) | 0.40 |  | 8 (14.6) | 5 (9.6) | 0.65 |  | 8 (14.3) | 5 (9.1) | 0.38 |  | 6 (10.9) | 4 (7.0) | 0.88 |  |
|  | 1 | 17 (28.8) | 17 (28.3) |  |  | 12 (21.8) | 14 (26.9) |  |  | 11 (19.6) | 17 (30.9) |  |  | 16 (29.1) | 18 (31.6) |  |  |
|  | 2 | 7 (11.9) | 12 (20.0) |  |  | 9 (16.4) | 9 (17.3) |  |  | 11 (19.6) | 11 (20.0) |  |  | 10 (18.2) | 14 (24.6) |  |  |
|  | 3 | 9 (15.3) | 17 (28.3) |  |  | 13 (23.6) | 19 (36.5) |  |  | 11 (19.6) | 14 (25.5) |  |  | 14 (25.5) | 13 (22.8) |  |  |
|  | 4 | 7 (11.9) | 2 (3.3) |  |  | 7 (12.7) | 2 (3.9) |  |  | 6 (10.7) | 5 (9.1) |  |  | 4 (7.3) | 6 (10.5) |  |  |
|  | 5 | 6 (10.2) | 5 (8.3) |  |  | 2 (3.6) | 1 (1.9) |  |  | 6 (10.7) | 1 (1.8) |  |  | 3 (5.5) | 1 (1.8) |  |  |
|  | 6 | 7 (11.9) | 0 (0.0) |  |  | 4 (7.3) | 2 (3.9) |  |  | 3 (5.4) | 2 (3.6) |  |  | 2 (3.6) | 1 (1.8) |  |  |
|  | 7 | 0 (0.0) | 2 (3.3) |  |  | 0 (0.0) | 0 (0.0) |  |  | 0 (0.0) | 0 (0.0) |  |  | 0 (0.0) | 0 (0.0) |  |  |
| Symptoms were rated on a 5-point scale from 0-4 for dryness and erythema and an 8-point scale from 0-7 for wrinkles. For dryness and erythema, 0 means "no symptoms," 4 means "significant symptoms," | | | | | | | | | | | | | | | |  |  |
| and for wrinkle severity, 0 means "no wrinkles" and 7 means "significant deep wrinkles. | | | | | | | | | | | |  |  |  |  |  |  |
| The Wilcoxon rank sum test was used to analyze differences between groups. No statistical significance was found between the two groups for three items. | | | | | | | | | | | | | | | | | |

| Table S3.VAS score for subjective skin symptoms. | | | | |
| --- | --- | --- | --- | --- |
|  |  | M-16V | Placebo | *P*-value |
| Overall skin condition | Baseline | 50.0 (35.0-62.5) | 52.0 (47.0 - 65.0) | 0.282 |
|  | Week 4 | 54.0 (49.0-74.0) | 60.5 (48.3 - 75.0) | 0.704 |
|  | Week 8 | 61.0 (52.0-75.0) | 64.0 (50.5 - 74.0) | 0.979 |
|  | Week 12 | 67.0 (51.5-78.0) | 65.0 (52.5 - 78.0) | 0.906 |
| Gloss | Baseline | 49.0 (35.0-53.5) | 49.0 (37.0 - 56.0) | 0.888 |
|  | Week 4 | 51.0 (48.0-65.8) | 53.5 (46.8 - 72.0) | 0.604 |
|  | Week 8 | 55.0 (49.0 - 73.0) | 58.0 (48.8 - 68.0) | 0.673 |
|  | Week 12 | 65.0 (50.0 - 76.3) | 59.5 (49.0 - 77.0) | 0.463 |
| Firmness | Baseline | 48.0 (29.0 - 53.5) | 44.5 (31.5 - 56.3) | 0.854 |
|  | Week 4 | 54.0 (47.5 - 65.5) | 56.0 (46.8 - 69.0) | 0.449 |
|  | Week 8 | 56.0 (50.0 - 72.0) | 56.5 (48.8 - 68.3) | 0.843 |
|  | Week 12 | 64.0 (51.0 - 75.0) | 57.5 (49.0 - 73.8) | 0.380 |
| Texture | Baseline | 44.0 (24.0 - 52.5) | 45.0 (31.5 - 54.0) | 0.390 |
|  | Week 4 | 51.5 (46.5 - 63.5) | 53.5 (46.0 - 74.0) | 0.546 |
|  | Week 8 | 55.0 (50.0 - 69.0) | 53.5 (47.0 - 68.0) | 0.577 |
|  | Week 12 | 60.0 (50.0 - 76.0) | 54.0 (46.5 - 76.0) | 0.276 |
| Dullness | Baseline | 36.0 (21.5 - 51.5) | 40.0 (30.5 - 50.3) | 0.389 |
|  | Week 4 | 49.5 (38.5 - 55.8) | 56.0 (44.5 - 69.5) | 0.036* |
|  | Week 8 | 55.0 (49.0 - 65.0) | 54.0 (48.0 - 68.0) | 0.573 |
|  | Week 12 | 55.0 (48.0 - 70.0) | 52.0 (45.3 - 67.8) | 0.731 |
| Spots | Baseline | 37.0 (20.5 - 49.5) | 40.0 (26.3 - 54.3) | 0.252 |
|  | Week 4 | 50.0 (37.0 - 57.5) | 54.0 (44.5 - 69.8) | 0.065 |
|  | Week 8 | 53.0 (47.0 - 64.0) | 53.0 (45.8 - 67.3) | 0.485 |
|  | Week 12 | 57.0 (48.0 - 72.0) | 52.5 (46.0 - 70.0) | 0.411 |
| Dark circles | Baseline | 47.0 (24.5 - 59.0) | 45.0 (30.0 - 55.0) | 0.942 |
|  | Week 4 | 52.0 (41.5 - 59.0) | 51.0 (42.0 - 74.0) | 0.582 |
|  | Week 8 | 53.0 (44.0 - 69.0) | 52.5 (47.8 - 68.3) | 0.685 |
|  | Week 12 | 57.0 (43.0 - 74.0) | 58.5 (46.0 - 73.0) | 0.973 |
| Redness | Baseline | 48.0 (35.5 - 69.5) | 55.0 (48.0 - 75.5) | 0.071 |
|  | Week 4 | 55.0 (51.0 - 69.0) | 60.0 (50.8 - 78.3) | 0.390 |
|  | Week 8 | 63.0 (50.0 - 75.0) | 65.0 (50.0 - 74.0) | 0.741 |
|  | Week 12 | 66.0 (50.0 - 79.0) | 66.5 (51.0 - 79.5) | 0.591 |
| Pores | Baseline | 42.0 (25.0 - 52.0) | 46.5 (25.8 - 56.3) | 0.289 |
|  | Week 4 | 53.0 (45.0 - 62.0) | 52.0 (45.0 - 76.0) | 0.547 |
|  | Week 8 | 56.0 (43.0 - 71.0) | 60.0 (51.5 - 72.5) | 0.369 |
|  | Week 12 | 59.0 (51.0 - 74.0) | 53.0 (46.4 - 77.8) | 0.460 |
| Dryness | Baseline | 44.0 (26.0 - 54.8) | 49.0 (37.0 - 58.5) | 0.363 |
|  | Week 4 | 51.0 (39.5 - 61.5) | 51.5 (36.0 - 65.3) | 0.803 |
|  | Week 8 | 52.0 (38.0 - 72.0) | 51.5 (39.0 - 67.8) | 0.956 |
|  | Week 12 | 66.0 (44.0 - 74.0) | 53.0 (38.0 - 73.0) | 0.129 |
| Wrinkles | Baseline | 43.0 (24.0 - 51.0) | 45.5 (28.3 - 56.0) | 0.419 |
|  | Week 4 | 53.0 (41.0 - 58.5) | 53.0 (42.3 - 66.5) | 0.600 |
|  | Week 8 | 53.0 (44.0 - 66.0) | 54.5 (42.8 - 73.3) | 0.415 |
|  | Week 12 | 62.0 (51.0 - 74.0) | 54.0 (44.0 - 75.0) | 0.302 |
| Tightness after washing the face | Baseline | 51.0 (33.5 - 76.0) | 51.0 (38.3 - 64.0) | 0.947 |
|  | Week 4 | 58.5 (51.0 - 71.8) | 56.0 (43.0 - 74.3) | 0.379 |
|  | Week 8 | 57.0 (48.0 - 74.0) | 59.5 (51.8 - 75.5) | 0.573 |
|  | Week 12 | 67.0 (51.0 - 79.0) | 60.0 (51.0 - 77.3) | 0.381 |
| Rough skin | Baseline | 51.0 (33.5 - 71.5) | 53.0 (47.3 - 64.0) | 0.470 |
|  | Week 4 | 60.0 (52.0 - 77.5) | 59.5 (45.0 - 74.5) | 0.545 |
|  | Week 8 | 54.0 (47.0 - 81.0) | 67.0 (51.0 - 79.5) | 0.272 |
|  | Week 12 | 68.0 (51.0 - 80.0) | 64.0 (51.3 - 80.0) | 0.935 |
| Lip condition | Baseline | 52.0 (39.5 - 71.0) | 53.0 (46.0 - 65.0) | 0.671 |
|  | Week 4 | 53.0 (44.5 - 67.8) | 58.5 (45.8 - 72.0) | 0.580 |
|  | Week 8 | 53.0 (38.0 - 68.0) | 56.5 (48.5 - 75.3) | 0.104 |
|  | Week 12 | 56.5 (46.75 - 72.5) | 54.0 (48.0 - 71.0) | 0.850 |
| Nail condition | Baseline | 52.0 (44.0 - 75.0) | 52.5 (42.0 - 65.3) | 0.636 |
|  | Week 4 | 53.0 (50.0 - 74.0) | 63.5 (50.0 - 72.5) | 0.651 |
|  | Week 8 | 55.0 (50.0 - 68.0) | 60.5 (50.8 - 75.3) | 0.304 |
|  | Week 12 | 64.0 (52.0 - 77.0) | 59.0 (50.0 - 76.0) | 0.685 |
| Hair condition | Baseline | 50.0 (38.5 - 63.0) | 51.0 (35.8 - 67.8) | 0.732 |
|  | Week 4 | 54.0 (51.0 - 69.5) | 63.0 (49.8 - 76.3) | 0.481 |
|  | Week 8 | 54.0 (50.0 - 69.0) | 67.5 (52.0 - 76.0) | 0.091 |
|  | Week 12 | 67.0 (52.0 - 76.0) | 60.0 (52.0 - 80.0) | 0.962 |
| Data are presented as the medians (interquartile ranges). A higher value for the VAS score indicates a better condition, while a lower value indicates a worse condition. | | | | |
| The VISIA total score was calculated by adding all eight items from brown spots to wrinkles for each subject. | | | | |
| The Wilcoxon rank sum test was used to analyze differences between groups. **P*<0.05 | | | |  |

| Table S4. Alpha diversity of fecal and skin microbiota and skin mycobiota in M-16V and placebo groups. | | | | | | | | |
| --- | --- | --- | --- | --- | --- | --- | --- | --- |
|  |  | week 0 | |  |  | week 12 | |  |
|  |  | M-16V | Placebo | *P*-value |  | M-16V | Placebo | *P*-value |
| **Fecal microbiota** | |  |  |  |  |  |  |  |
|  | Shannon | 4.97 (4.54-5.27) | 4.79 (4.54-5.11) | 0.169 |  | 5.02 (4.68-5.18) | 4.89 (4.53-5.15) | 0.192 |
|  | Chao1 | 66.0 (57.5-85.6) | 62.5 (54.8-71.1) | 0.091 |  | 68.1 (56.4-78.6) | 64.7 (54.3-72.8) | 0.342 |
|  | Evenness | 0.83 (0.77-0.85) | 0.81 (0.78-0.85) | 0.599 |  | 0.82 (0.79-0.84) | 0.81 (0.78-0.84) | 0.496 |
|  | Faith_pd | 7.46 (6.73-8.88) | 7.22 (6.63-8.23) | 0.336 |  | 7.69 (6.63-8.63) | 7.47 (6.69-8.25) | 0.454 |
|  | Obsereved_OTUs | 66.0 (57.5-84.5) | 62.5 (54.8-71.0) | 0.096 |  | 67.0 (56.0-78.3) | 63.0 (54.3-72.0) | 0.338 |
| **Skin microbiota** | |  |  |  |  |  |  |  |
|  | Shannon | 2.77 (1.67-4.53) | 3.20 (1.80-4.87) | 0.490 |  | 2.94 (2.08-5.31) | 3.61 (2.21-5.21) | 0.434 |
|  | Chao1 | 248.0 (125.0-367.0) | 237.5 (163.0-404.8) | 0.483 |  | 259.0 (169.0-398.5) | 251.5 (186.3-404.4) | 0.783 |
|  | Evenness | 0.37 (0.25-0.54) | 0.42 (0.26-0.61) | 0.425 |  | 0.39 (0.31-0.60) | 0.45 (0.32-0.64) | 0.454 |
|  | Faith_pd | 31.0 (22.5-42.2) | 32.2 (24.9-42.9) | 0.595 |  | 33.2 (27.4-45.3) | 34.8 (26.2-49.2) | 0.892 |
|  | Obsereved_OTUs | 183.0 (91.5-299.5) | 181.5 (128.5-325.8) | 0.579 |  | 195.5 (127.5-338.0) | 204.0 (140.5-332.0) | 0.869 |
| **Skin fungal mycobiota** | |  |  |  |  |  |  |  |
|  | Shannon | 2.63 (1.94-4.03) | 2.47 (1.96-3.54) | 0.159 |  | 3.44 (2.62-4.23) | 3.02 (2.00-3.88) | 0.101 |
|  | Chao1 | 72.3 (44.6-103.5) | 60.6 (36.5-84.1) | 0.080 |  | 77.5 (52.8-98.6) | 58.8 (39.5-99.0) | 0.098 |
|  | Obsereved_OTUs | 52.0 (37.0-75.0) | 49.5 (30.3-67.5) | 0.190 |  | 62.5 (45.3-78.0) | 44.5 (31.3-67.8) | 0.061 |
| Data are presented as the medians (interquartile ranges). | | | | | | | | |
| The Wilcoxon rank sum test was used to analyze differences between groups. | | | | | | | | |

| Table S5. Composition of the fecal microbiota in M-16V and placebo groups. | | | | | | | | | |
| --- | --- | --- | --- | --- | --- | --- | --- | --- | --- |
|  |  |  | week 0 | |  |  | week 12 | |  |
|  |  |  | M-16V | Placebo | *P*-value |  | M-16V | Placebo | *P*-value |
| **Total population** | | |  |  |  |  |  |  |  |
|  | **Phylum** | |  |  |  |  |  |  |  |
|  |  | Actinobacteria | 5.78 (2.21-11.3) | 6.79 (2.94-10.8) | 0.562 |  | 4.98 (2.07-9.90) | 5.77 (1.77-12.2) | 0.579 |
|  |  | Bacteroidetes | 33.8 (24.2-41.3) | 33.4 (28.0-40.9) | 0.817 |  | 34.2 (27.3-39.7) | 33.8 (28.4-40.7) | 0.742 |
|  |  | Firmicutes | 47.4 (40.7-54.9) | 47.4 (38.5-57.6) | 0.894 |  | 51.4 (41.6-57.9) | 44.6 (37.7-57.2) | 0.120 |
|  |  | Proteobacteria | 5.58 (2.13-16.1) | 3.78 (1.59-12.0) | 0.191 |  | 2.72 (1.36-11.9) | 4.04 (1.65-10.9) | 0.563 |
|  | **Genus** | |  |  |  |  |  |  |  |
|  |  | *Bacteroides* | 27.0 (16.9-34.3) | 24.7 (19.1-33.1) | 0.791 |  | 24.6 (17.8-34.3) | 25.9 (16.2-35.0) | 0.962 |
|  |  | *Bifidobacterium* | 5.41 (1.47-10.9) | 6.43 (2.45-10.6) | 0.527 |  | 4.92 (1.95-9.05) | 5.22 (1.77-11.9) | 0.594 |
|  |  | *Blautia* | 3.92 (2.21-5.67) | 3.29 (1.38-5.72) | 0.423 |  | 4.89 (2.59-6.91) | 3.92 (1.85-4.99) | 0.041* |
|  |  | *Coprococcus* | 1.14 (0.41-2.74) | 0.88 (0.21-2.15) | 0.321 |  | 1.27 (0.49-2.50) | 1.32 (0.65-2.33) | 0.914 |
|  |  | *Escherichia* | 1.06 (0.03-3.80) | 0.55 (0.00-3.34) | 0.50 |  | 0.31 (0.00-1.81) | 0.44 (0.00-4.51) | 0.413 |
|  |  | *Faecalibacterium* | 10.1 (5.15-20.0) | 10.1 (2.84-20.4) | 0.663 |  | 14.0 (6.97-22.4) | 10.0 (5.71-19.5) | 0.351 |
|  |  | *Parabacteroides* | 1.56 (0.73-2.83) | 1.57 (0.53-2.62) | 0.646 |  | 1.48 (0.82-2.71) | 1.06 (0.39-2.45) | 0.274 |
|  |  | *Roseburia* | 1.93 (0.10-3.70) | 0.50 (0.00-2.63) | 0.107 |  | 1.91 (0.26-5.34) | 1.42 (0.50-4.58) | 0.807 |
|  |  | *Ruminococcus* | 2.10 (0.39-3.99) | 2.14 (0.26-6.05) | 0.644 |  | 2.70 (0.46-5.12) | 2.72 (0.52-5.79) | 0.838 |
| **≧50 years population** | | |  |  |  |  |  |  |  |
|  | **Phylum** | |  |  |  |  |  |  |  |
|  |  | Actinobacteria | 5.40 (3.09-8.54) | 6.19 (2.65-10.1) | 0.884 |  | 4.29 (1.77-7.28) | 4.75 (1.23-10.3) | 0.837 |
|  |  | Bacteroidetes | 34.2 (23.8-41.8) | 29.8 (24.4-40.0) | 0.734 |  | 34.4 (27.5-40.0) | 33.2 (27.6-43.8) | 0.821 |
|  |  | Firmicutes | 47.4 (43.9-53.7) | 52.9 (41.8-62.9) | 0.516 |  | 51.0 (43.5-61.2) | 41.3 (35.8-55.8) | 0.030* |
|  |  | Proteobacteria | 3.79 (1.69-10.6) | 2.64 (1.25-5.23) | 0.128 |  | 2.92 (1.77-8.48) | 6.38 (2.30-15.8) | 0.201 |
|  | **Genus** | |  |  |  |  |  |  |  |
|  |  | *Bacteroides* | 27.2 (15.7-35.9) | 22.8 (14.9-28.5) | 0.197 |  | 29.3 (17.9-33.8) | 22.5 (15.8-34.2) | 0.398 |
|  |  | *Bifidobacterium* | 5.03 (1.88-7.47) | 5.57 (1.51-8.86) | 0.771 |  | 4.22 (1.41-7.25) | 4.51 (1.21-10.3) | 0.869 |
|  |  | *Blautia* | 4.15 (2.53-5.49) | 1.91 (1.24-4.68) | 0.114 |  | 5.94 (2.46-7.91) | 3.52 (1.34-4.76) | 0.018* |
|  |  | *Coprococcus* | 1.31 (0.71-2.11) | 1.19 (0.33-2.09) | 0.528 |  | 1.27 (0.50-2.90) | 1.20 (0.27-2.84) | 0.726 |
|  |  | *Escherichia* | 0.65 (0.00-2.21) | 0.36 (0.00-0.88) | 0.355 |  | 0.46 (0.00-1.88) | 0.82 (0.00-5.49) | 0.360 |
|  |  | *Faecalibacterium* | 9.73 (5.70-18.8) | 9.08 (2.85-19.4) | 0.485 |  | 15.7 (6.79-22.9) | 9.12 (5.45-15.7) | 0.149 |
|  |  | *Parabacteroides* | 1.46 (0.76-2.67) | 1.26 (0.53-2.27) | 0.347 |  | 1.48 (0.87-2.89) | 0.91 (0.47-1.54) | 0.073 |
|  |  | *Roseburia* | 1.50 (0.21-2.39) | 0.43 (0.00-8.26) | 0.687 |  | 1.67 (0.30-4.48) | 1.34 (0.24-5.52) | 0.835 |
|  |  | *Ruminococcus* | 0.84 (0.38-5.26) | 1.87 (0.00-6.10) | 0.930 |  | 2.11 (0.66-4.45) | 1.62 (0.11-5.03) | 0.536 |
| **＜50 years population** | | |  |  |  |  |  |  |  |
|  | **Phylum** | |  |  |  |  |  |  |  |
|  |  | Actinobacteria | 7.28 (1.88-12.0) | 7.65 (3.57-13.9) | 0.479 |  | 5.43 (2.19-12.7) | 6.78 (2.45-14.3) | 0.644 |
|  |  | Bacteroidetes | 33.5 (24.4-40.6) | 35.1 (29.4-41.9) | 0.494 |  | 34.2 (27.1-39.6) | 34.0 (29.5-40.3) | 0.778 |
|  |  | Firmicutes | 47.1 (39.0-55.3) | 45.0 (37.0-56.1) | 0.525 |  | 51.6 (39.2-57.5) | 46.7 (38.4-58.9) | 0.768 |
|  |  | Proteobacteria | 5.76 (2.91-17.6) | 5.84 (2.08-13.8) | 0.657 |  | 2.66 (1.20-14.1) | 3.41 (1.54-7.67) | 0.878 |
|  | **Genus** | |  |  |  |  |  |  |  |
|  |  | *Bacteroides* | 26.4 (19.4-32.5) | 29.1 (21.0-34.8) | 0.414 |  | 22.6 (17.6-34.3) | 29.4 (17.8-35.0) | 0.342 |
|  |  | *Bifidobacterium* | 7.23 (1.37-11.8) | 7.29 (3.57-13.2) | 0.442 |  | 5.18 (2.14-11.3) | 6.29 (2.23-14.0) | 0.599 |
|  |  | *Blautia* | 3.59 (1.95-5.84) | 3.54 (2.14-6.02) | 0.829 |  | 4.67 (3.15-5.59) | 4.19 (2.41-5.61) | 0.644 |
|  |  | *Coprococcus* | 0.84 (0.25-3.60) | 0.72 (0.09-2.24) | 0.363 |  | 1.25 (0.49-2.43) | 1.42 (0.87-1.81) | 0.873 |
|  |  | *Escherichia* | 1.16 (0.13-3.97) | 1.40 (0.24-4.08) | 0.819 |  | 0.27 (0.00-1.03) | 0.33 (0.00-3.11) | 0.676 |
|  |  | *Faecalibacterium* | 10.5 (4.90-20.1) | 10.9 (4.13-21.0) | 0.962 |  | 12.1 (7.31-22.0) | 13.2 (5.76-21.2) | 1.000 |
|  |  | *Parabacteroides* | 1.62 (0.77-2.90) | 1.71 (0.60-2.81) | 0.815 |  | 1.44 (0.56-2.27) | 1.35 (0.35-2.58) | 0.898 |
|  |  | *Roseburia* | 2.02 (0.05-4.70) | 0.56 (0.00-2.11) | 0.071 |  | 1.91 (0.26-5.94) | 1.42 (0.61-4.24) | 0.908 |
|  |  | *Ruminococcus* | 2.16 (0.47-3.92) | 2.42 (0.39-5.99) | 0.573 |  | 3.43 (0.46-5.54) | 2.88 (0.95-6.92) | 0.496 |
| Data are presented as the medians (interquartile ranges) for taxa with a median relative abundance greater than 1% in at least one group. | | | | | | | | | |
| The Wilcoxon rank sum test was used to analyze differences between groups. **P*<0.05 | | | | | | | | | |

| Table S6. Composition of the skin microbiota and mycobiota in M-16V and placebo groups. | | | | | | | | | |
| --- | --- | --- | --- | --- | --- | --- | --- | --- | --- |
|  |  |  | week 0 | |  |  | week 12 | |  |
|  |  |  | M-16V | Placebo | *P*-value |  | M-16V | Placebo | *P*-value |
| **Skin microbiota** | | |  |  |  |  |  |  |  |
|  | **Phylum** | |  |  |  |  |  |  |  |
|  |  | Actinobacteriota | 68.0 (44.1-87.5) | 65.0 (46.2-90.7) | 0.621 |  | 64.1 (30.2-84.7) | 64.6 (43.1-86.5) | 0.773 |
|  |  | Bacteroidota | 1.06 (0.34-2.96) | 0.77 (0.29-1.98) | 0.381 |  | 1.30 (0.46-3.01) | 1.54 (0.48-2.88) | 0.968 |
|  |  | Firmicutes_D | 5.78 (2.68-15.7) | 5.31 (2.83-9.16) | 0.313 |  | 5.10 (3.52-11.3) | 6.75 (5.41-15.1) | 0.241 |
|  |  | Proteobacteria | 15.1 (5.02-27.1) | 14.1 (3.08-33.3) | 0.460 |  | 17.2 (6.88-32.6) | 11.9 (4.24-26.8) | 0.475 |
|  | **Genus** | |  |  |  |  |  |  |  |
|  |  | *Corynebacterium* | 1.71 (0.76-3.79) | 1.73 (0.96-3.84) | 0.758 |  | 1.61 (0.72-3.79) | 1.47 (0.69-2.81) | 0.576 |
|  |  | *Cutibacterium* | 54.3 (28.4-80.0) | 51.5 (27.5-83.7) | 0.915 |  | 44.1 (17.3-77.6) | 55.8 (28.3-81.2) | 0.771 |
|  |  | *Staphylococcus* | 1.85 (0.72-3.12) | 1.39 (0.72-2.68) | 0.313 |  | 1.54 (0.48-3.54) | 1.31 (0.66-3.14) | 0.336 |
|  |  | *Streptococcus* | 1.46 (0.43-5.74) | 1.75 (0.62-4.02) | 0.992 |  | 2.76 (1.27-5.57) | 3.83 (0.85-6.89) | 0.893 |
| **Skin mycobiota** | | |  |  |  |  |  |  |  |
|  | **Phylum** | |  |  |  |  |  |  |  |
|  |  | Ascomycota | 6.61 (2.77-22.1) | 6.56 (2.72-17.7) | 0.614 |  | 13.2 (5.52-30.4) | 7.67 (2.18-21.9) | 0.065 |
|  |  | Basidiomycota | 87.7 (66.8-94.7) | 87.5 (69.5-95.6) | 0.659 |  | 70.5 (53.6-89.0) | 78.6 (59.9-95.5) | 0.174 |
|  | **Genus** | |  |  |  |  |  |  |  |
|  |  | *Aspergillus* | 1.07 (0.35-2.33) | 0.74 (0.11-1.75) | 0.113 |  | 0.71 (0.38-4.44) | 0.87 (0.07-2.04) | 0.161 |
|  |  | *Malassezia* | 75.5 (43.6-86.4) | 78.8 (50.7-91.8) | 0.298 |  | 59.3 (36.0-80.5) | 60.6 (43.7-88.3) | 0.332 |
| Data are presented as the medians (interquartile ranges) for taxa with a median relative abundance greater than 1% | | | | | | | | | |
| in at least one group. The Wilcoxon rank sum test was used to analyze differences between groups. | | | | | | | | | |
| No statistical significance was found between the two groups. | | | | | | | | | |

| Table S7.VISIA score for each skin indicator in a group with low defecation frequency. | | | | |  |  |  |  |  |
| --- | --- | --- | --- | --- | --- | --- | --- | --- | --- |
|  |  | M-16V | Placebo | Between-groups | Within-group p-value | | Difference between changes from baseline | | |
|  |  |  |  | *P*-value | M-16V | Placebo | M-16V | Placebo | *P*-value (between-groups) |
| Brown spots | Baseline | 22.3 (17.6-25.7) | 17.3 (12.8-20.5) | 0.011* | - | - | - | - | - |
|  | Week 4 | 20.3 (16.8-25.2) | 16.9 (14.3-23.6) | 0.216 | 0.191 | 0.615 | -0.29 (1.38-0.67) | 0.14 (-1.36-1.85) | 0.240 |
|  | Week 8 | 20.3 (16.5-26.2) | 18.1 (14.0-23.7) | 0.110 | 0.278 | 0.484 | -0.62 (-2.08-0.93) | 0.04 (-1.13-2.21) | 0.230 |
|  | Week 12 | 23.0 (17.6-26.1) | 18.0 (15.1-20.4) | 0.092 | 0.569 | 0.741 | -0.42 (-2.06-1.20) | 0.11 (-0.97-1.36) | 0.500 |
| Pores | Baseline | 18.6 (14.3-22.3) | 12.4 (8.83-24.8) | 0.181 | - | - | - | - | - |
|  | Week 4 | 19.1 (13.4-22.7) | 14.1 (8.72-26.6) | 0.279 | 0.211 | 0.808 | -1.81 (-4.25-0.81) | 0.04 (-3.41-1.59) | 0.338 |
|  | Week 8 | 16.5 (12.8-24.2) | 16.9 (9.26-23.9) | 0.732 | 0.501 | 0.951 | -0.36 (-2.69-0.89) | -0.09 (-2.58-2.33) | 0.568 |
|  | Week 12 | 13.5 (12.0-19.6) | 14.5 (7.02-27.1) | 0.713 | 0.044* | 0.434 | -2.17 (-7.39-0.88) | 0.21 (-1.03-3.49) | 0.04* |
| Porphyrins | Baseline | 2.83 (2.09-4.28) | 4.40 (2.81-7.31) | 0.087 | - | - | - | - | - |
|  | Week 4 | 2.89 (2.62-4.43) | 5.72 (3.18-7.15) | 0.138 | 0.910 | 0.833 | -0.02 (-0.68-0.70) | -0.10 (-0.79-0.94) | 0.951 |
|  | Week 8 | 3.28 (2.20-4.34) | 5.49 (3.27-6.42) | 0.130 | 0.569 | 0.605 | 0.21 (-0.45-0.75) | -0.19 (-0.76-0.69) | 0.441 |
|  | Week 12 | 3.21 (2.05-4.67) | 4.46 (2.49-6.06) | 0.232 | 0.501 | 0.434 | 0.23 (-0.41-0.76) | -0.31 (-1.33-0.60) | 0.283 |
| Red areas | Baseline | 9.03 (8.63-10.7) | 9.19 (7.52-9.96) | 0.323 | - | - | - | - | - |
|  | Week 4 | 9.20 (8.68-9.84) | 9.11 (7.91-9.91) | 0.536 | 0.650 | 0.935 | -0.15 (-0.67-0.56) | 0.04 (-0.92-0.67) | 0.688 |
|  | Week 8 | 9.23 (8.27-10.9) | 9.17 (7.92-11.3) | 0.886 | 0.877 | 0.301 | 0.32 (-1.34-0.97) | 0.32 (-0.66-1.88) | 0.458 |
|  | Week 12 | 9.03 (7.96-10.3) | 9.72 (8.49-11.2) | 0.374 | 0.535 | 0.039* | -0.11 (-0.95-0.57) | 0.28 (-0.02-1.49) | 0.098 |
| Spots | Baseline | 27.1 (22.8-31.1) | 24.2 (21.3-26.4) | 0.149 | - | - | - | - | - |
|  | Week 4 | 29.5 (23.7-32.8) | 21.7 (18.4-29.7) | 0.038* | 0.036* | 0.101 | 1.64 (0.21-2.62) | -1.58 (-3.51-1.17) | 0.024* |
|  | Week 8 | 26.2 (22.3-34.2) | 22.9 (19.8-28.0) | 0.145 | 0.679 | 0.761 | 0.33 (-1.80-2.18) | 0.06 (-2.71-1.77) | 0.689 |
|  | Week 12 | 27.7 (23.1-34.2) | 22.0 (19.8-25.6) | 0.066 | 0.163 | 0.339 | 1.45 (-0.32-2.70) | 0.05 (-2.61-0.84) | 0.061 |
| Texture | Baseline | 5.90 (3.17-8.16) | 5.03 (2.77-7.14) | 0.423 | - | - | - | - | - |
|  | Week 4 | 6.54 (2.55-8.60) | 4.91 (2.43-6.85) | 0.307 | 0.570 | 0.961 | -0.14 (-0.73-0.45) | -0.02 (-0.41-0.60) | 0.665 |
|  | Week 8 | 5.32 (3.14-8.07) | 4.98 (2.51-7.27) | 0.408 | 0.030* | 0.903 | -0.58 (-0.95-0.25) | 0.21 (-0.64-0.70) | 0.104 |
|  | Week 12 | 5.55 (2.70-7.77) | 4.78 (2.86-6.72) | 0.902 | 0.006* | 0.520 | -0.74 (-1.31--0.14) | 0.45 (-0.72-0.70) | 0.014* |
| UV spots | Baseline | 22.4 (19.1-28.4) | 19.3 (14.3-25.0) | 0.200 | - | - | - | - | - |
|  | Week 4 | 23.0 (18.8-27.0) | 23.9 (18.0-26.3) | 0.926 | 0.865 | 0.277 | 0.61 (-1.31-1.10) | 0.50 (-1.16-2.61) | 0.516 |
|  | Week 8 | 24.4 (19.3-28.7) | 24.9 (17.2-26.8) | 0.511 | 0.109 | 0.059 | 0.33 (-0.24-2.13) | 0.78 (-0.40-3.06) | 0.753 |
|  | Week 12 | 23.4 (21.1-27.4) | 24.6 (15.0-26.8) | 0.408 | 0.07 | 0.092 | 1.20 (0.56-2.27) | 0.64 (-0.58-2.85) | 0.581 |
| Wrinkles | Baseline | 34.2 (27.5-43.9) | 40.0 (22.5-45.1) | 0.915 | - | - | - | - | - |
|  | Week 4 | 38.2 (29.4-47.9) | 36.1 (33.5-48.1) | 0.829 | 0.125 | 0.050 | 2.74 (-0.73-5.63) | 4.34 (-0.59-7.26) | 0.599 |
|  | Week 8 | 38.4 (28.7-47.3) | 37.2 (30.0-46.3) | 0.954 | 0.109 | 0.260 | 2.19 (-1.89-4.67) | 2.61 (-4.05-8.89) | 0.977 |
|  | Week 12 | 39.7 (31.3-43.9) | 43.4 (32.7-48.7) | 0.520 | 0.030* | 0.030* | 3.62 (-0.64-6.61) | 4.74 (-2.65-10.7) | 0.624 |
| Total score | Baseline | 148.0 (136.6-169.9) | 134.3 (113.6-150.5) | 0.082 | - | - | - | - | - |
|  | Week 4 | 153.4 (132.4-166.7) | 144.7 (116.6-161.3) | 0.386 | 0.460 | 0.168 | 3.17 (-4.95-9.46) | 5.90 (-5.05-9.32) | 0.734 |
|  | Week 8 | 148.6 (133.3-170.4) | 145.8 (120.4-161.8) | 0.376 | 0.408 | 0.201 | 1.75 (-2.35-8.38) | 5.73 (-4.03-13.2) | 0.530 |
|  | Week 12 | 150.3 (136.1-164.8) | 144.8 (114.1-157.8) | 0.312 | 0.918 | 0.030* | -0.98 (-8.42-7.35) | 9.08 (-1.11-14.9) | 0.070 |
| Data are presented as the medians (interquartile ranges). A higher value for the VISIA score indicates a worse condition, while a lower value indicates a better condition. | | | | | | | | | |
| The VISIA total score was calculated by adding all eight items from brown spots to wrinkles for each subject. | | | | | | | | | |
| The Wilcoxon rank sum test was used to analyze differences between groups. | | | | |  |  |  |  |  |
| Within-group p-value for each grpup was calculated by comparing against baseline using the Wilcoxon signed rank test. | | | | | | | |  |  |
| The group with low defecation frequency refers to subjects with 5 or fewer defecations per week (M-16V: n=16, Placebo: n=25). | | | | | | | | | |

| Table S8. Composition of the fecal microbiota with low defecation frequency in M-16V and placebo groups. | | | | | | | | |
| --- | --- | --- | --- | --- | --- | --- | --- | --- |
|  |  | week 0 | |  |  | week 12 | |  |
|  |  | M-16V | Placebo | *P*-value |  | M-16V | Placebo | *P*-value |
| **Phylum** | |  |  |  |  |  |  |  |
|  | Actinobacteria | 3.55 (1.89-6.54) | 8.49 (6.04-14.9) | 0.028* |  | 3.84 (1.94-5.05) | 5.15 (1.17-13.0) | 0.250 |
|  | Bacteroidetes | 41.3 (35.4-46.6) | 31.8 (27.4-40.8) | 0.082 |  | 30.9 (27.5-36.3) | 31.5 (28.5-39.5) | 0.362 |
|  | Firmicutes | 45.6 (39.1-51.3) | 44.2 (32.3-55.4) | 0.593 |  | 57.1 (51.5-63.9) | 42.7 (38.4-57.0) | 0.018* |
|  | Proteobacteria | 4.49 (1.81-14.2) | 5.84 (2.23-13.8) | 0.575 |  | 3.45 (1.77-6.18) | 7.02 (2.20-15.2) | 0.250 |
| **Genus** | |  |  |  |  |  |  |  |
|  | *Bacteroides* | 34.1 (27.0-36.3) | 22.8 (15.3-31.2) | 0.037* |  | 24.5 (16.7-30.1) | 25.3 (15.7-34.0) | 0.893 |
|  | *Bifidobacterium* | 3.20 (1.25-5.74) | 7.79 (5.59-13.6) | 0.011* |  | 3.28 (1.75-4.80) | 4.86 (1.27-12.5) | 0.226 |
|  | *Blautia* | 3.87 (1.51-5.52) | 3.54 (1.78-4.73) | 0.852 |  | 4.96 (2.36-5.95) | 4.00 (2.41-4.88) | 0.521 |
|  | *Coprococcus* | 1.46 (0.74-2.78) | 1.12 (0.31-2.59) | 0.538 |  | 0.90 (0.54-2.45) | 1.60 (0.99-2.29) | 0.174 |
|  | *Escherichia* | 1.42 (0.34-4.21) | 2.16 (0.36-10.6) | 0.437 |  | 0.00 (0.00-0.69) | 1.02 (0.11-6.28) | 0.021* |
|  | *Faecalibacterium* | 8.48 (5.31-14.5) | 7.01 (1.96-16.8) | 0.438 |  | 20.5 (11.9-27.8) | 8.12 (3.47-16.7) | 0.043* |
|  | *Parabacteroides* | 1.88 (1.26-4.36) | 2.19 (0.79-2.98) | 0.504 |  | 1.72 (0.99-2.66) | 1.33 (0.74-3.44) | 0.560 |
|  | *Roseburia* | 1.33 (0.20-3.42) | 0.00 (0.00-0.56) | 0.031* |  | 0.96 (0.18-4.49) | 0.74 (0.29-3.10) | 0.730 |
|  | *Ruminococcus* | 2.39 (0.58-8.59) | 3.37 (1.19-6.77) | 0.567 |  | 4.23 (2.10-9.43) | 4.48 (1.85-8.09) | 0.988 |
| Data are presented as the medians (interquartile ranges) for taxa with a median relative abundance greater than 1% in at least one group. | | | | | | | | |
| A group with low defecation frequency refers to a group with a defecation frequency of 5 or less times/week at 0 week. | | | | | | | | |
| The Wilcoxon rank sum test was used to analyze differences between groups. **P*<0.05 | | | | | | | | |
