# Supplementary figures and images for: Imaging and Microorganism Analyses of the Effects of Oral Bifidobacterium breve Intake on Facial Skin in Females: A Randomized, Double-Blind, Placebo-Controlled Study
Source: Nutrients. 2025 Sep 17;17(18):2976. doi: 10.3390/nu17182976 (PMC12472256; doi:10.3390/nu17182976)

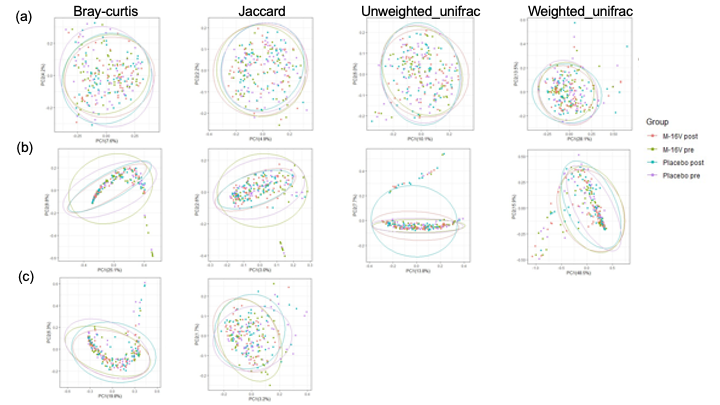

Supplement: Supplementary file 1 [file nutrients-17-02976-s001.zip › Supfiles/Figsup1.tiff]
